# Abnormal expression of CD96 on natural killer cell in peripheral blood of patients with chronic obstructive pulmonary disease

**DOI:** 10.1111/crj.13523

**Published:** 2022-07-22

**Authors:** Xiaomin Zhao, Xiaowen Feng, Pengcheng Liu, Jing Ye, Rui Tao, Renming Li, Bing Shen, Xiaoming Zhang, Xuefu Wang, Dahai Zhao

**Affiliations:** ^1^ Department of Respiratory and Critical Care Medicine, the Second Affiliated Hospital Anhui Medical University Hefei China; ^2^ School of Pharmacy Anhui Medical University Hefei China; ^3^ School of Basic Medicine Anhui Medical University Hefei China

**Keywords:** CD16, CD96, COPD, LUNG, NK cell

## Abstract

Natural killer (NK) cells are regarded as the host's first line of defense against viral infection. Moreover, the involvement of NK cells in chronic obstructive pulmonary disease (COPD) has been documented. However, the specific mechanism and biological changes of NK cells in COPD development have not been determined. In this study, we extracted NK cells from the peripheral blood of 18 COPD patients who were recovering from an acute exacerbation and 45 healthy donors (HDs), then we labeled NK cells with different antibodies and analyzed with flow cytometry. The data showed that the frequencies of total NK cells in the peripheral blood of COPD patients were lower compared with HDs. Moreover, the inhibitory receptors on NK cells expressed higher levels and the expression of activating receptors were generally low. Importantly, both the expression levels of CD96 in NK cells and the frequencies of CD96^+^ NK cells were significantly upregulated in COPD patients. These findings suggest that surface receptor CD96 from NK cells may be a risk factor in the evolution of COPD.

## Graphical abstract

The study shows that the expression levels of NK cell surface receptors were abnormal in chronic obstructive pulmonary disease (COPD) patients compared with healthy controls, especially inhibitory receptors CD96. These findings suggest that surface receptor CD96 from NK cells may be a risk factor in the evolution of COPD.

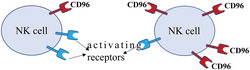

**Abbreviations**
COPD: Chronic obstructive pulmonary disease
FITC: Fluorescein Isothiocyanate
HDs: Healthy donors
INF: Interferon
NK: Natural killer
Siglec‐7: Sialic acid‐binding Ig‐like lectin 7
Siglec‐9: Sialic acid‐binding Ig‐like lectin 9
TIGIT: T cell immunoreceptor with Ig and ITIM domains
TIM‐3: T cell immunoglobulin and mucin domain‐3
TNF: Tumor necrosis factor

## 1 INTRODUCTION

Chronic obstructive pulmonary disease (COPD) is the third leading cause of morbidity and death, imposing a significant socioeconomic burden globally.^1^ Pathogenesis of COPD is associated with chronic inflammation of the respiratory tract and is characterized by persistent airflow limitation.^2^ Risk factors of COPD include smoking, pollution, and genetic determinants, with smoking being the main risk factor. Smoking triggers chronic lung inflammation, impairs the functioning of the lung barrier, and reduces immune defense mechanisms, leading to increased susceptibility to respiratory infections.^3^ NK cells form the first line of the innate immune system to against diseases, including infections and malignant neoplasms.^4^As an important effector arm of the innate immune system, NK cells constitute about 10% of the lymphocytes in human PB. In addition, NK cells play an important role in supplying interferon (IFN)‐γ and tumor necrosis factor (TNF)‐α.^5^ NK cells act not only through direct pathways to mediate tissue immune injury but also through indirect pathways.^6^ Moreover, NK cells can limit or exacerbate immune responses by regulating cells engaged in reciprocal interactions with immune cells, such as T cells, dendritic cells, and endothelial cells. Activation of NK cells results from the integration of multiple activating and inhibitory signals that vary depending on the nature of the interacting cells.^7^ The activation of an NK cell to kill a targeted cell is tightly regulated by signals which are received via NK cell surface receptors, including inhibitory and activating receptors.^8^ With different functions and maturation statuses, these receptors on NK cells are divided into two main subsets: CD56^bright^CD16^−^ and CD56^dim^CD16^+^.^9^ Nearly 90% of the peripheral blood NK cells are the CD56^dim^CD16^+^ NK cells, which contain high levels of granzymes, perforin, and cytolytic granules. These NK cells can also produce a large amount of cytokines, including IFN‐γ and TNF‐α.^10^ CD56 (also called neural cell adhesion molecule), as an important NK cell antigen belonging to the immunoglobulin superfamily, mediates homophilic adhesion.^11^ In humans, NK cells expressing high levels of CD56, the predominant subset in lymph nodes, produce high cytokine.^12^ CD96 as an inhibitory receptor expressed on the surface NK cells plays an important inhibitory role in immune function.^13^ It can regulate NK cell effector function and metastasis and can be a novel target for cancer immunotherapy in recent studies.^14^ No study to date, however, has shown a specific relationship between the expression of NK cell surface receptors and COPD.

Management of COPD remains in the symptomatic treatment stage, with no targeted treatment available. By deciphering the functions of these receptors in the PB of COPD patients will increase our understanding of the mechanisms associated with NK cell exhaustion and inform COPD therapy. Enhancing our understanding of the biology of NK cells in the PB of COPD patients may facilitate the development of NK cell‐based immunotherapy, individualized treatment of COPD.

Therefore, in the present study, we assessed that the expression levels of either activating or inhibitory receptors on NK cells were different between patients with COPD and healthy donor control participants especially the expression levels of CD96.

## 2 MATERIALS AND METHODS

### 2.1 Participants

Peripheral blood (5 ml) was obtained using standard procedures from 45 healthy donors (mean age, 60 ± 5 years) and from 18 patients with COPD (mean age, 60 ± 5 years). (Table 1)The samples of healthy group were collected from people for physical examination in medical examination center. The samples of COPD group were collected on the day of discharge of patients who stopped intravenous drugs for more than 3 days, and airway reaction symptoms were significantly relieved.

**TABLE 1:** Clinical and demographic characteristics of the study populations

Characteristics	Control group	COPD group	*p*‐value
Male gender	30 (66.67)	11 (61.11)	0.772
Age, years	60.04 ± 3.36	61.78 ± 2.22	0.052
BMI, kg/m^2^	20.07 ± 1.46	21.55 ± 1.84	0.053
Ever smoker	28(62.22)	12(66.67)	0.781
FEV_1_ (% predicted)	111.00 ± 6.19	57.48 ± 6.53	<0.0001
FEV_1_/FVC (%)	83.84 ± 2.65	56.59 ± 4.26	<0.0001
CAT score	0 ± 0.00	6.28 ± 1.48	<0.0001

*Note*: Data presented as mean ± SD. The figures in brackets are % for gender and smokers.

Abbreviations: CAT, COPD assessment test; COPD, chronic obstructive pulmonary disease; FEV1, forced expiratory volume in 1 s; FVC, forced vital capacity.

### 2.2 Antibodies

We used labeled antibodies to identify the following receptors or markers of NK cells: CD3 (FITC conjugated), CD56 (APC‐Cy7 conjugated), T cell immunoreceptor with Ig and ITIM domains (TIGIT) (PerCp‐Cy5.5 conjugated), CD16 (Alexa 700 conjugated), sialic acid‐binding Ig‐like lectin 7 (Siglec‐7) (PE conjugated), Siglec‐9 (APC conjugated), T cell immunoglobulin and mucin domain‐3 (TIM‐3) (Qdot 605 conjugated), PD‐1 (V450 conjugated), CD73 (PE conjugated), CD39 (APC conjugated), NKp44 (PE‐Cy7 conjugated), NKG2D (Qdot 605 conjugated), NKp46 (PerCp‐Cy5.5 conjugated), and CD96 (PE‐Cy7 conjugated).

### 2.3 Experimental procedure

#### 2.3.1 Lymphocyte extraction

Peripheral blood was centrifuged at 1500 × *g* for 10 min; the resulting supernatant was removed and stored at −80°C. The pelleted cells were resuspended in 5 ml of 1 × phosphate‐buffered saline (PBS) and then layered on human peripheral blood lymphocyte isolation solution (5 ml). The suspension was centrifuged for 30 min at 800 × *g* rising 6 up/2 down 24°C. The extracted NK cells were suspended in 10 ml of 1 × PBS, centrifuged at 400 × *g* 10 up/ 10 down/10 24°C for 10 min. The previous step was then repeated.

#### 2.3.2 Experimental groups, counting, and antibody incubation

After centrifugation, 1 ml of RPMI 1640 sterile solution was added to the pelleted cells for use in the experiments. Each of these experimental samples was divided into four groups: control (ISO), NK1, NK2, and NK3. To 100 μl of each group sample, 20 μl of mouse serum was added and mixed, and the samples were placed at 4°C for 20 min.

A 10‐μl sample was removed from each group and used for cell counting.

For the ISO group, antibodies to CD3 and CD56 were used. For the NK1 group, antibodies to CD3, CD56, S‐7, S‐9, CD16, TIGIT, TIM‐3, and PD‐1 were used. For the NK2 group, antibodies to CD3, CD56, CD‐73, CD39, NKp44, NKG2D, and NKp46 were used. For the NK3 group, antibodies to CD3, CD56, and CD96 were used. All procedures were conducted to avoid light. We used 10 U of antibody for every 1 × 10^7^ cells. All antibody incubations were carried out at 4°C for 30 min.

#### 2.3.3 Flow cytometry

Each sample was resuspended in 2 ml of 1 × PBS and centrifuged at 400 × *g* for 10 min. The resulting supernatant was removed, and the resuspension and centrifugation step was repeated. Flow cytometry was used to detect, identify, and count the cells. FlowJO software was used to analyze the flow cytometry data.

#### 2.3.4 Statistical analysis

Significant differences among groups were identified by *t*‐test or by one‐way analysis of variance, as appropriate. Differences between means were considered statistically significant at *P* < 0.05. Data were analyzed using Prism software, version 8 (GraphPad).

## 3 RESULTS

### 3.1 Abnormal numbers and frequencies of NK cells observed in the PB of COPD patients

**FIGURE 1:**
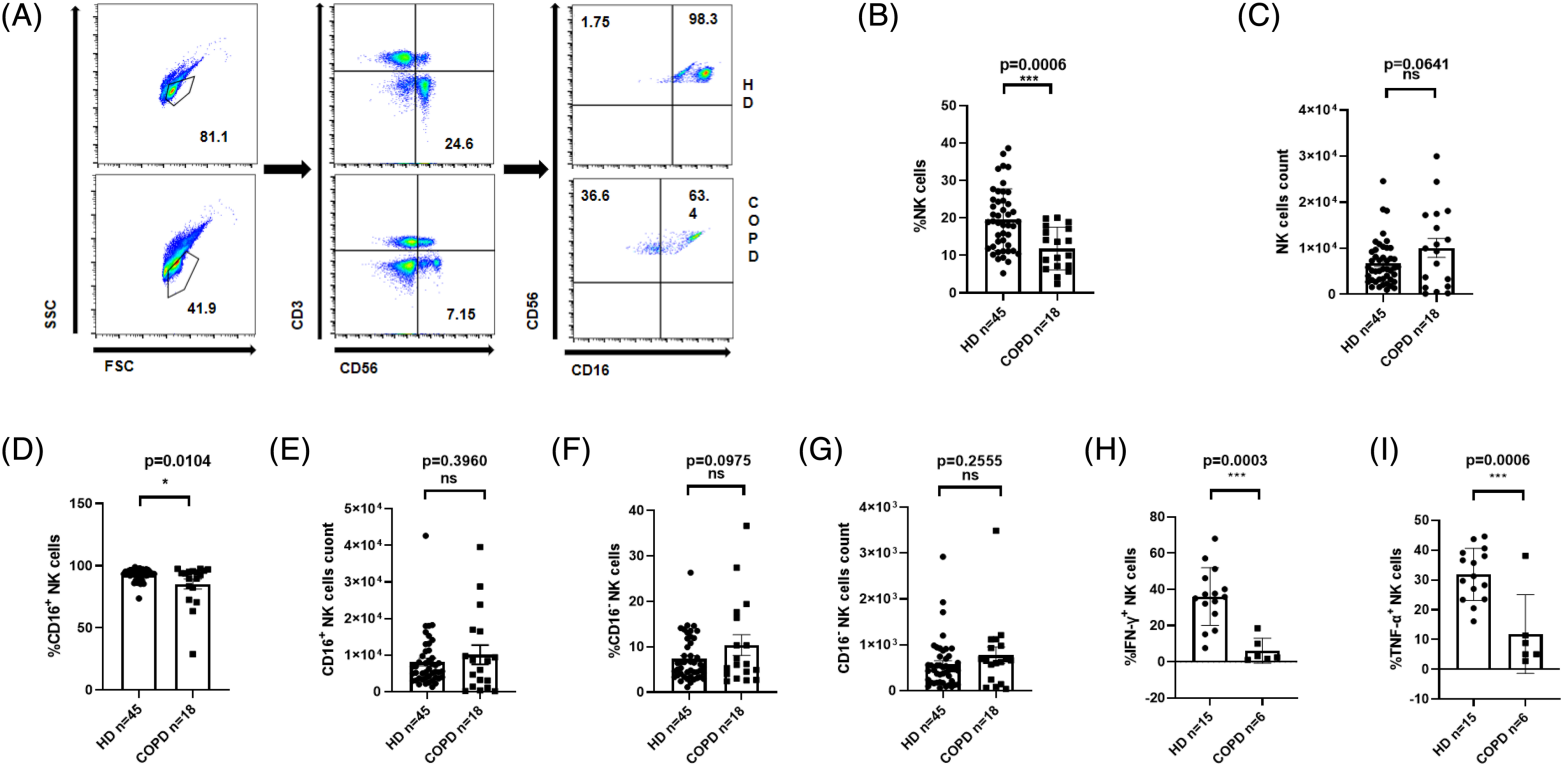
(A) Gating strategy used for the cytometric analysis of natural killer (NK) cell subsets in PBMC samples. (B, D, F) Frequencies of NK cells within the lymphocyte population (B), CD16^+^ NK cell subset (D), and CD16^−^ NK cell subset (F) in total NK cells from peripheral blood of patients with chronic obstructive pulmonary disease (COPD) and healthy donors (HDs). (C, E, and G) Numbers of NK cells (C), CD16^+^ NK cells (E), and CD16^−^ NK cells (G) in the peripheral blood of patients with COPD and HDs. (H, I) the proportion of IFN‐γ^+^ NK cells (H) and TNF‐α^+^ NK cells (I) in patients with COPD and HDs. Data are shown as means ± SDs. *P* < 0.05 considered statistically significant; *P < 0.05; ***P < 0.001; ns, not significant

The expression levels of superficial receptors on NK cells in PB derived from COPD patients and HDs were assessed, and the potential for the use of these receptors as potential targets for COPD therapy was explored. The samples of COPD group were collected from the patients hospitalized for an average of 13.5 days (the range is 10–16 days) and stopped intravenous drugs for more than 3 days. The blood was drawn on the day of discharge, and the airway reaction symptoms of patients were significantly relieved for an average of 5.67 days (the range is 4–8 days). CD3^−^CD56^+^ NK cells in PB were identified within the lymphocyte gate and then subdivided into CD56^dim^CD16^+^ and CD56^bright^CD16^−^ NK subsets (Figure 1A). The results show that the frequency (or percentage) of total NK cells in the PB of HDs was higher compared with COPD patients (Figure 1B). However, the absolute number of NK cells in the PB did not differ between COPD patients and HDs (Figure 1C). Similarly, the absolute numbers of both CD56^dim^CD16^+^ and CD56^bright^CD16^−^ NK cell subsets did not differ between COPD patients and HDs (Figure 1E,G). The proportion of the CD56^dim^CD16^+^ NK subset was lower in COPD patients compared with HDs, whereas the proportion of the CD56^bright^CD16^−^ NK subset showed no marked difference between the COPD and control groups (Figure 1D,F). Moreover, the NK cells from COPD patients displayed decreased IFN‐γ production (Figure 1H,I). Taken together, our data revealed a decrease in the frequency (percentage), number, and function of NK cells in patients with COPD.

### 3.2 Abnormal expression of activating and inhibitory receptors on NK cells in patients with COPD

**FIGURE 2:**
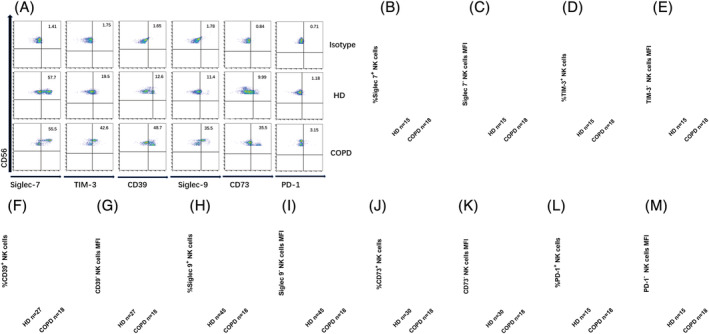
Abnormal expression of inhibitory receptors on the surface natural killer (NK cells) in peripheral blood of patients with chronic obstructive pulmonary disease (COPD). (A) Representations of Siglec‐7, TIM‐3, CD39, Siglec‐9, CD73, and PD‐1 expression on NK cells in patients with COPD and in healthy donors (HDs). (B, D, F, H, J, L) Proportion of Siglec‐7^+^ (B), TIM‐3^+^ (D), CD39^+^ (F), Siglec‐9^+^ (H), CD73^+^ (J), or PD‐1^+^ (L) NK cells in patients with COPD and in HDs. (C, E, G, I, K, M) Mean fluorescence intensity (MFI) ratios of Siglec‐7^−^ (C), TIM‐3^−^ (E), CD39^−^ (G), Siglec‐9^−^ (I), CD73^−^ (K), or PD‐1^−^ (M) NK cells. MFI ratio of X = (X − MFI)/(isotype−MFI). Data are shown as means ± SDs. *P* < 0.05 considered statistically significant; *P < 0.05; **P < 0.01; ****P < 0.0001; ns, not significant

**FIGURE 3:**
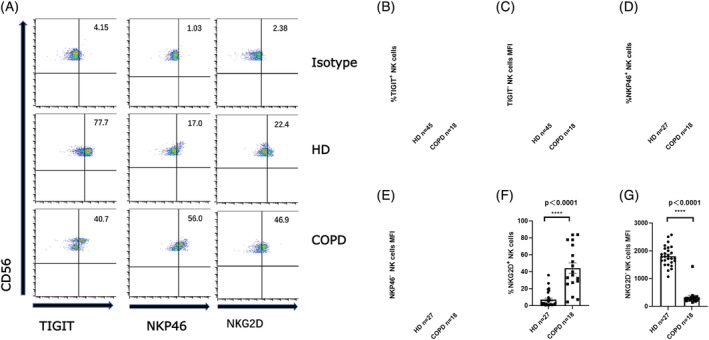
(A) Representations of TIGIT, NKP46, and NKG2D expression on natural killer (NK) cells in patients with chronic obstructive pulmonary disease (COPD) and healthy donors (HDs). (B, D, F) Proportion of TIGIT^+^ (B), NKP46^+^ (D), or NKG2D^+^ (F) NK cells in patients with COPD and HDs. (C, E, G) Mean fluorescence intensity (MFI) ratios of TIGIT^−^ (C), NKP46^−^ (E), NKG2D^−^, or (G) NK cells. MFI ratio of X = (X − MFI)/(isotype−MFI). Data are shown as means ± SDs. *P* < 0.05 considered statistically significant; **P < 0.01; ***P < 0.001; ****P < 0.0001; ns, not significant

The activity of NK cells is regulated by activating and inhibitory receptors on the surface. We investigated the expression characteristics of the activating and inhibitory receptors on NK cells in PB of COPD patients compared with donors. We found that the expression frequencies of TIM‐3^+^, CD73^+^, PD‐1^+^, Siglec‐9^+^, and CD39^+^ NK cells in COPD patients were upregulated compared with HDs (Figure 2D–M). However, the frequency of Siglec‐7^+^ NK cells was lower in COPD (Figure 2B). No significant difference in the frequencies of TIGIT^+^ NK cells was observed between the two groups (Figure 3B). Moreover, the mean fluorescence intensity (MFI) level of TIGIT^dim^ expression on NK cells in COPD grope was significantly lower than that in HDs (Figure 3C). The frequency of NKp46^+^ NK cells was slightly higher in the patient group although frequencies were low in both groups (Figure 3D). Taken together with the aforementioned data showing that the expression of CD16^+^ NK cells is abnormal in COPD group compared with controls (Figure 1D), our results indicated that the expression levels of activating and inhibitory NK cell surface receptors were abnormal in COPD patients, suggesting NK cell dysfunction. Our results indicated that the proportion of inhibitory receptors on NK cells in the PB of COPD group was generally increased compared with HDs (Figure 2).

### 3.3 Expression of inhibitory receptor CD96 is increased in the peripheral blood of COPD patients

**FIGURE 4:**
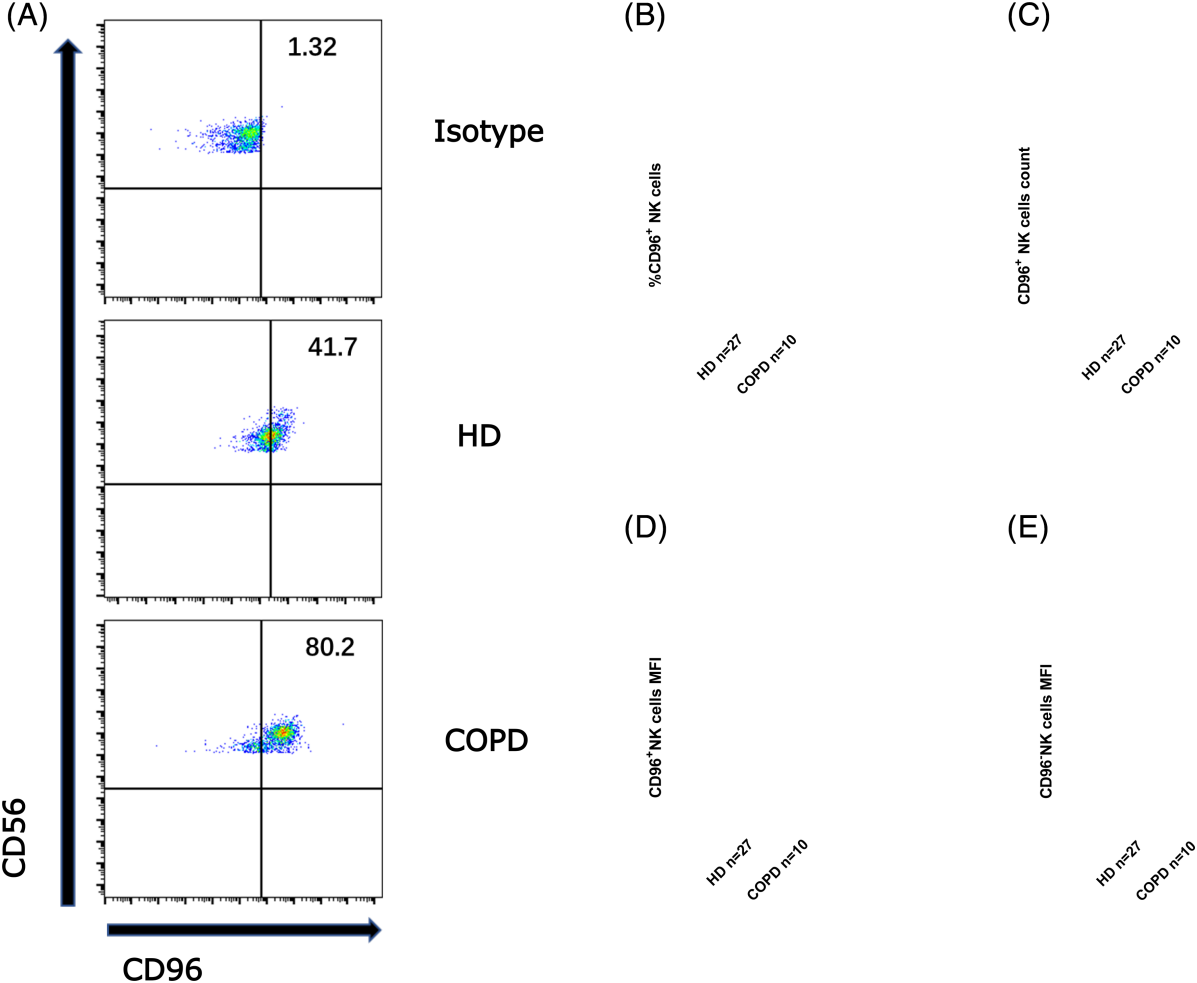
(A) Representations of CD96^+^ natural killer (NK) cell expression in peripheral blood of patients with chronic obstructive pulmonary disease (COPD) and of healthy donors (HDs). (B) The proportion of CD96^+^ NK cells in patients and HDs. (C) Numbers of CD96^+^ NK cells in peripheral blood of patients and HDs. (D, E) Mean fluorescence intensity (MFI) ratios of CD96^+^ (D) or CD96^−^ (E) NK cells. MFI ratio of X = (X − MFI)/(isotype−MFI). Data are shown as means ± SDs. *P* < 0.05 considered statistically significant; *P < 0.05; ***P < 0.001; ****P < 0.0001

The frequency of CD96^+^ NK cells in the PB of COPD patients was markedly upregulated compared with HDs (Figure 4B). CD96 is a transmembrane glycoprotein immunoglobulin superfamily receptor expressed on T cells and NK cells.^15^ The main ligand for CD96 is CD155, to which it binds with an affinity stronger than CD226 but weaker than TIGIT.^13^ Our results showed that the absolute number of NK cells in the PB of COPD patients did not change significantly compared with HDs, whereas the number of CD96^+^ NK cells in COPD patients was upregulated compared with HDs (Figure 4C). However, the MFI level of CD96 expression on NK cells was significantly lower in the patient group than in HDs (Figure 4D,E). Thus, our data indicated that the expression of CD96 was higher on NK cells in the PB of COPD patients.

## 4 DISCUSSION

Previous studies have shown that NK cells play a key role in liver disease and acute lung injury.^16^ It has been reported that NK cells also play an important role in COPD.^9^ In our present study, we found a functional defect in the NK cells of patients with COPD and the frequency of total NK cells in PB was lower in COPD patients compared with HDs. However, we observed no marked difference in the absolute number of NK cells in PB between these patients and HDs. These findings are similar to those reported previously.^17^ By contrast, Urbanowicz et al. showed that NK cells were reduced and natural killer T cells in terms of both numbers and cytotoxicity in the PB of COPD patients, with no difference in producing cytokine in these cells between patients and HDs.^18^ Although the numbers and function of NK cells in COPD patients are controversial, our study supports the former conclusion. Because NK cells are considered the first line of defense against infection in the innate immune system, our data suggest that the functions of NK cells are impaired in COPD patients, potentially explaining why these patients are easier to get an infection.

The triggering of NK cell activation typically involves two modes: “missing‐self” recognition and “induced‐self” recognition. Missing‐self recognition occurs when the target cells display lower or even absent surface expression of major histocompatibility complex class I molecules, which are generally associated with viral infection or cellular transformation.^9^ This decreased expression leads to weakening of the inhibitory signal, resulting in activation of NK cells. Activated NK cells release cytokines, such as IFN‐γ and TNF‐α.^19^ Activated NK cells function in various environments, mainly through cytotoxicity and cytokine production.^9^ Some studies have shown that NK cells are the predominate producers of IFN‐γ in response to viral ligands and that this production is enhanced after cigarette smoke exposure.^20^ It has been widely reported that long‐term cigarette smoke exposure, which eventually alters the function of the lung barrier and reduces immune defense mechanisms, is the major cause of COPD.^21^ The cytotoxic attacks of NK cell are immediate, do not require prior antigen‐priming, and are instead orchestrated uniquely by myriad receptors with activating or inhibitory functions.^4^ In our study, the frequencies of CD16^+^ NK cells were high in both patients and healthy donors although the frequency was slightly higher in the patient group. CD16 expression is typically assessed along with CD56 expression, with CD16^+^ NK cells considered to be cytotoxic.^22^ The CD16 receptor mediates binding to antibodies through the Fc portion of IgG, triggering antibody‐dependent cellular cytotoxicity, but it can also mediate IFN‐γ, TNF‐α, and chemokine production.^2^, ^23^, ^24^

The function of NK cell is determined by the integration of signals arising from the engagement of different NK receptors with specific ligands on potential target cells.^25^ Our study found that the expression levels of the activated receptors were upregulated, suggesting that the NK cells are highly cytotoxic. Cytotoxic NK cells play an essential role in the clearance of viral infections and malignant cells.^22^ Thus, our results revealed a functional defect in the NK cells of COPD patients, with the expression levels of CD16 in COPD group suggesting that other factors led to the dysfunction.

When we assessed the expression of other receptors, we found that the frequencies of NKG2D^+^ and NKp46^+^ NK cells in PB of COPD patients were upregulated compared with HDs. This result suggests that these receptors may play a role in promoting the occurrence of inflammation and that NKG2D on NK cells is necessary for enhanced pulmonary inflammation and airway injury following influenza infection in COPD.^20^

Functional defects in NK cells involve increased expression of inhibitory receptors. We found that the expression of some inhibitory receptors, including TIM‐3, CD73, PD‐1, Siglec‐9, CD39, and CD96, was increased on NK cells in peripheral blood of patients with COPD, especially the CD96 receptor. CD96 and CD226 receptors share the ligand CD155, and the CD96 receptor inhibits the function of NK cells by competitively binding CD155 with the CD226 receptor.^26^ Studies have shown that the expression of the inhibitory receptor CD96 is also upregulated on tumor NK cells.^15^ CD96 deficiency may enhance the immunomodulatory effect of NK cells. Studies have shown that targeted inhibition of CD96 reduces the pathological damage in lung tissue of mice infected with *Chlamydia muridarum* and reduces the pulmonary bacterial load.^26^ The finding in our study of the significant increase in inhibitory receptor CD96 in COPD patients suggests that CD96 may play an important role in NK cell functional impairment. In addition, the expression of CD96 was generally high in patients with COPD. Thus, we suggest that CD96 may be considered as a target receptor in the development of treatments for patients with COPD. Therapeutic blockade of CD96 in tumor metastasis models confirmed its role as a checkpoint receptor on NK cells.^15^ Existing treatments for COPD are largely symptomatic, and the only approved anti‐inflammatory medication, corticosteroids, has no proven modifying disease‐modifying effect.^27^ The high expression of inhibitory receptors in patients with COPD provides a new therapeutic direction, namely, to target NK cell surface receptors and enhance their role in immune regulation.

For patients with COPD, infection is an important cause of acute exacerbation of COPD. A minimum of 4 weeks is required after an exacerbation before a patient can be considered stable. Due to the impact of the COVID‐19 pandemic, it is difficult to collect stationary samples in the community. A significant limitation was that the patients were studied while recovering from an acute exacerbation. We selected samples of COPD patients who hospitalized for an average of 13.5 days and collected samples on the day of discharge to reduce the influence of infectious factors and intravenous medication on experimental results. The biology of CD96 is poorly studied, and more work is required to understand how much our results would have been influenced by the effect of the acute inflammation with the exacerbation.

## 5 CONCLUSION

Our study showed that the expression of activating and inhibitory receptors on the surface of NK cells in peripheral blood of patients with COPD differs significantly from that in healthy people. The expression level of CD96 on peripheral blood NK cells in particular was significantly increased in these patients. Our findings serve to deepen our understanding of CD96^+^ NK cells. The study is an exploratory experiment and only reveals the possible mechanism. It could be a foundation for the study of the correlation between pulmonary function and CD96 expression in patients with COPD.

## CONFLICT OF INTEREST

The authors report no conflicts of interest in this work.

## ETHICS STATEMENT

Approval was obtained from The Second Hospital of Anhui Medical University Ethics Committee for the study (serial number: YX2021‐081).

## AUTHOR CONTRIBUTIONS

Xiaomin Zhao, Xuefu Wang, and Dahai Zhao conceived and designed the research; Xiaomin Zhao, Xiaowen Feng, Pengcheng Liu, Rui Tao, and Renming Li performed the experiments; Xiaomin Zhao, Xiaowen Feng, Pengcheng Liu, Rui Tao, and Renming Li analyzed the data; Xiaomin Zhao, Xiaowen Feng, Pengcheng Liu, Rui Tao, and Renming Li interpreted the results of the experiments; Xiaomin Zhao, Xiaowen Feng, Pengcheng Liu, Jing Ye, Rui Tao, and Renming Li prepared the figures; Jing Ye, Bing Shen, and Xiaoming Zhang collected the clinical data; Xiaomin Zhao, Xiaowen Feng, and Pengcheng Liu drafted the manuscript; Xiaomin Zhao, Xiaowen Feng, Pengcheng Liu, Xuefu Wang, and Dahai Zhao edited and revised the manuscript. All authors have read and approved the final manuscript.

## Data Availability

The data that support the findings of this study are available from the corresponding author upon reasonable request.
